# 'One patient amongst many': a qualitative analysis of intensive care unit patients' experiences of transferring to the general ward

**DOI:** 10.1186/cc6795

**Published:** 2008-02-22

**Authors:** Kate Field, Suman Prinjha, Kathy Rowan

**Affiliations:** 1 DIPEx Research Group, Department of Primary Health, University of Oxford, Old Road Campus, Oxford OX3 7LF, UK; 2 Intensive Care National Audit and Research Centre (ICNARC), Tavistock House, Tavistock Square, London WC1H 9HR, UK

## Abstract

**Introduction:**

Many patients experience 'relocation stress' when they are transferred from an intensive care unit (ICU) to step-down (high dependency) or general ward care, and much has been written about the psychological causes. This qualitative analysis of in-depth, narrative interviews with former ICU patients explores and examines patients' accounts in order to identify additional causes of relocation stress.

**Methods:**

Forty former ICU patients were recruited throughout the UK, using maximum variation sampling, to achieve a broad range of experiences of intensive care. Interviews in people's homes were recorded on video and audio equipment as part of a study for the Database of Personal Experiences of Health and Illness (DIPEx) web resource. All interviews were transcribed, checked and returned to respondents. For this report, a qualitative thematic analysis was used to explore experiences of transfer.

**Results:**

We found that most people experienced relocation distress not only because of physical and emotional difficulties relating to their illness and treatment and the inevitable anxiety resulting from leaving a protected environment, but also from concrete, practical causes. These included specific concerns about communication, feeding, nursing care and support, as well as ward organization and environment. Written excerpts from the interviews and two video excerpts taken from the DIPEx website illustrate our findings.

**Conclusion:**

We conclude that there are several aspects of care that deserve further examination by researchers and service providers, and that not all of the factors associated with relocation stress should be seen as an inevitable consequence of the psychological adjustment involved in transfer from an ICU.

## Introduction

Patients admitted to intensive care units (ICUs) need constant, close monitoring and specialist nursing to keep them alive [1]. Usually one nurse looks after one patient. Patients discharged from ICUs to general wards can be vulnerable, and if they are discharged prematurely they may quickly become worse [2-4]. Patients in ICUs can lose up to 2% of muscle mass for each day of illness, and some patients may take a year to recover completely [5]. After discharge from the ICU, patients may experience altered sleep patterns, anxiety, depression [6,7], disorientation, mood changes [8], and lapses of memory and concentration [9,10]. Some former ICU patients continue to have hallucinations, nightmares, or delusions even after their discharge from hospital [11].

During the past 10 years, many practical strategies have been applied to prevent adverse events and to help patients to adapt to the general ward following the 'security blanket' of the ICU. These include step-down or high dependency units, outreach services [12], critical care nurse consultants [13], specialist information packs and ward follow-up [14-16]. In 2005, the UK Critical Care Stakeholder Forum emphasized the need for continuity of care and suggested that 'care bundles' be established [17]. Studies from Australia and New Zealand, as well as the UK, have also examined general ward and critical care nurses' feelings about transfer stress and identified that many general ward staff feel inadequately prepared to care for ICU patients and that handovers were often rushed [18-20]. Another study of ward staff in the UK found that they wanted more training to help them to cope with patients transferred from ICU [21].

Qualitative and mixed methods research has helped to draw attention to the emotional distress experienced by many patients leaving ICU, as well as that of carers and close family members [22-24]. A recent literature review of relocation stress concluded that future research should focus on the causes rather than the categories of distress, and that more studies based on UK data were needed [25]. Recently, the UK National Institute for Health and Clinical Excellence (NICE) published guidelines for Management of the Acutely Ill Patients, and acknowledged that despite all of the developments already outlined, patients in the National Health Service (NHS) are not yet experiencing 'a smooth, planned transfer' from ICU to the general ward and recommended that patient transfers should be standardized [26].

Our analysis of patient experiences of transferring from ICU to the general ward adds rich insights into how patients experience transfer, and adds weight to NICE's calls for the standardization of patient transfers and increased training for ward staff. Our national study differs from many previous qualitative studies in ICUs which were conducted by health-care professionals in their own work setting. Recruitment to the study was based on a maximum variation sample to include a broad range of patient experiences reflecting not only different reasons for ICU admission but also diversity between respondents in terms of age, ethnicity, length of time since in ICU, occupational background and geographical location. SP, an experienced qualitative researcher from a nonclinical, social science background, interviewed respondents in their own homes. The deliberate lack of connection with the clinical environment encourages patients to speak more freely about their experiences, positive and negative.

This study analyses narrative interviews with people who had been transferred to a general ward to 'unpack' the meanings and causes of relocation stress in the accounts of former ICU patients. The data show that the cause of this stress is not an inevitable part of transfer from a relatively cocooned environment.

## Materials and methods

### The DIPEx Project

Database of Personal Experiences of Health and Illness (DIPEx) is an ongoing research project about people's experiences of health and illness that is based at the Department of Primary Care, University of Oxford. Currently, members of the DIPEx research team have completed 37 collections, including two projects about patients and carers' experiences of intensive care. All DIPEx projects are conducted with multicentre research ethics committee approval and based on maximum variation samples of around 40 to 50 narrative interviews. DIPEx interviews are conducted one-to-one in the respondent's home by an experienced senior qualitative researcher. All of the interviews are digitally recorded (video or audio) and professionally transcribed. DIPEx publishes its analysis and findings on its website [27], which is written primarily for a lay audience, and is also being used in health-care and inter-professional medical education. The wider aims of DIPEx are described in more detail elsewhere and on the website [28-30].

### The sample

Maximum variation samples are used in qualitative interview studies to ensure that a broad range of participants and experiences is included, not to be numerically representative. This means that the study may be generalizable in terms of the themes and issues that it identifies, but analysis should steer clear of relative frequencies, which are likely to mislead [31,32]. The maximum variation sample was used in this context to ensure that a broad range of patient experiences were captured. We had additional guidance in the parameters of the sample from an expert advisory panel of patient representatives, researchers and intensive care clinicians [33] who advised us, for example, to include the experiences of men and women admitted to intensive care for emergency and elective admissions as well as those who had experienced different lengths of stay.

Reasons for emergency admission included pneumonia, pancreatitis, head injury, other accidents, bowel perforation and surgical complications. Reasons for elective admission included surgery for various cancers and heart conditions. The sample also included the perspectives of patients from different social and occupational backgrounds, as well as those treated in different parts of the UK. The purpose of interviewing patients from throughout the UK is not to effect a detailed comparison of the impact of different hospital facilities on patients but to ensure that the sample includes many different experiences.

All but six of the 40 respondents had been admitted to ICU as emergencies, and it is their experiences that are the focus of this paper, because they would have had no prior warning of or preparation for ICU or the general ward. All were interviewed during 2005 to 2006 and they had been admitted to ICUs between 1994 and 2005 (Table 1).

**Table 1:** Participants and their length of stay in the ICU and the general ward: 34 participants who transferred to high dependency unit/general ward

Interview number	Age at interview (years)	Sex	Length of ICU/HDU stay^a^	Length of ward stay^a^
IC01	41	Female	ICU: 11 days	2 weeks
IC02	60	Male	ICU: about 1 month	4 days
IC03	66	Male	ICU: 5 weeks	5 weeks
IC04	46	Female	ICU: about 5 weeks/HDU: 1 week	Discharged after 1 week in HDU
IC05	40	Female	ICU: 22 days/HDU: 10 days	Just over a week
IC06	35	Female	ICU: 3 weeks/HDU: 36 hours	5 weeks
IC07	60	Male	ICU: 7 months	Ward: 1 month/home: awaiting rehabiliatation
IC08	50	Male	ICU: 10 days	Readmitted in 2006 and died
IC09	60	Male	ICU: 3 days	10 days
IC10	76	Female	ICU: about a week	1 week
IC12	23	Female	ICU: 21 days/HDU: 2 weeks	2 weeks
IC14	35	Female	ICU: 2 days	2 days, discharged herself
IC15	38	Female	ICU: 1 month, admitted three times in 2004	Several months on and off
IC16	67	Male	ICU: 8 weeks	3 weeks
IC17	30	Male	ICU: 12 days	2 weeks
IC18	62	Male	ICU: 18 days	5 weeks
IC21	72	Male	ICU: about 4 weeks	1 week
IC22	71	Male	ICU: 17 days	A few days
IC23	54	Male	ICU: 17 days	2 weeks
IC24	44	Female	ICU: 5 weeks	2 weeks
IC25	45	Male	ICU: 4 weeks	2 weeks
IC26	47	Male	ICU: 30 days	8 days
IC27	68	Male	ICU: 2 weeks/HDU: 2 weeks	Ward: 6 weeks/rehabilitation: 2 weeks
IC28	46	Male	ICU: 7 weeks	4 weeks
IC29	47	Female	ICU: 2 weeks/HDU: 1 day	1 week
IC30	55	Female	ICU: 6 days/HDU: 5 days	Discharged after HDU
IC31	71	Male	ICU: 2 weeks	3 weeks
IC32	57	Female	ICU: 29 days	Ward: 6 weeks/rehabilitation: 6 weeks
IC34	37	Male	ICU: 30 days total, admitted twice	Several months
IC35	33	Male	ICU: 17 days	Ward: 1 week/rehabilitation: 3 months
IC36	67	Female	ICU: unsure how long	Ward: unsure how long
IC37	58	Male	ICU: 9 days	16 days
IC38	55	Female	ICU: 1 week	2 weeks
IC39	56	Male	ICU: 4 days	3 days

^a^Respondents did not always know whether/when they had moved from intensive care unit (ICU) care to step-down or high-dependency unit (HDU) care. Some did not know or could not remember how long they had spent on a ward.

### Interviews

We used narrative interviews to elicit people's stories and perspectives of being in intensive care. Semistructured questions were used when necessary to ensure that everyone was given the opportunity to discuss anticipated and newly emerging themes. Each interview lasted up to 2 hours, and interviewing continued until data saturation was reached and nothing new was being added to the data or to the analytic categories [34]. Interviews were digitally recorded on video with consent from respondents and professionally transcribed. Each respondent received his or her own copy of the transcript for checking and validation. Once a final copy of the transcript had been agreed, he or she assigned copyright of the interview to DIPEx for use on the website and for research, publication, teaching and broadcasting.

### Analysis

Two researchers (SP and KF) scrutinized the data and constructed a coding framework. Interviews were systematically coded using a modified grounded theory approach so that data were explored for themes that were known in the literature as well as emergent themes. The experience of transfer stress was an anticipated theme (that is, it already existed in the qualitative literature about intensive care). N6 software was used to facilitate a comparison of themes across the entire dataset and also to highlight unusual ('deviant') cases. (QSR N6 software produced by QSR International Ltd, Doncaster, Victoria, Australia. QSR International is an international company that produces software packages. N6 is specficially designed to help researchers code and compare non-numerical unstructured data. Further details of methods, and the full range of findings from the DIPEx research, are available on the website.

## Results

Thirty-four people described what had happened to them when they left ICU, what they thought about their care and treatment, and they discussed the effect that their experience of transfer had on them and their families.

Respondents may not have been aware of specific constraints on the NHS, but most people understood and accepted that levels of care in ICU and the general ward differed. The majority said they had not received appropriate levels of care on the general ward or that they did not receive the kind of care that they had expected. Several people believed that their short-term recovery had been jeopardized as a result. Because we do not have access to patients' medical records, we are unable to validate their beliefs of how ill they were or how well they were recovering at the point of transfer or afterward. As outlined previously, the aim of DIPEx research is to collect and analyze patients' perceptions of events, and is not to prove or disprove their experiences with external clinical evidence, which would not in any case be available to us.

A 54-year-old man, who went onto the general ward for 2 weeks, expressed a widely held view that the NHS and its staff were dedicated and over-worked:

'They can't do enough for you [later in interview] They deserve everything, they don't deserve the bad publicity, the NHS. I've said you can't knock it for what they've done, for what they try to do. I think the people that, you know, sort of knock them ought to sit back and you know see how hard they work. Because when you're laid in bed you can actually see what the staff have to do and cope with. But they must be dedicated to what they do, to want to do it.' (IC23; man aged 54 years, treated in ICU for colorectal cancer complications.)

Several people, including a woman who had been an ICU nurse, realized that levels of care would differ between ICU and the general ward, and accepted that care on the general ward depended on the availability of resources:

'... you'd just have to wait but that's just the ward isn't it? Most wards are like that, they haven't got the staff that they've got on the intensive care unit, they've not got the resources and you haven't got your own nurse. But in a way you need that because you need to start to do more for yourself; simple things were hard to do, for example, going to the toilet or having a wash. You realize that you've got to get up and do it yourself, and I'd even have to wait for painkillers. But that's nobody's fault, that's just how it is.' (IC01; woman aged 41 years, in intensive care because of complications during pregnancy.)

For those who had not already talked about transferring from ICU to the general ward in the narrative section of the interview, an open question, 'What did you feel about your transfer from ICU to the general ward?', was asked. Many respondents became animated as they replied to this question; responses were usually lengthy. (For example a man aged 66 years who had spent 5 weeks in the ICU and 5 weeks on the ward spoke for 25 minutes without a break about his experiences on the ward.) Several people became emotional as they recollected what had happened to them and described the experience of transfer using words/phrases such as the following: 'it was a complete shock' (IC34); 'an awful experience to be honest ... we were both in tears constantly' (IC35); 'I felt very neglected' (IC29); 'from a state of nothingness ... became a state of utter confusion' (IC27); and 'it was a bit of a distressing experience' (IC10). The specific causes of people's distress at the time of their transfer from ICU and how those causes were attributed are shown on Table 2. We discuss our findings on patients' perceptions of care on the general ward under two overarching themes: care and support, and ward organization and environment.

**Table 2:** Causes of distress after transfer from the ICU to the general ward

Causes of distress		Respondents' attribution of the cause of their distress		
Instances of poor basic care	Instances where ward environment and organization inappropriate for recent ICU patients	Lack of resources in NHS	Lack of training	Inadequate handover from ICU

Left in chair by bed for 12 hours newly on ward Being left on commode on ward with diarrhoea and vomiting Being given food and buzzer when too weak to use arms Not being given pain relief when required Feeling 'smelly' after 2 days on ward with no washing Being bathed in tepid water and left undried Buzzer left answered for 'what seemed like hours' No help offered when profuse sweating started No action taken when he went from being extremely hot to cold and shivering Water in jug by bedside but not poured into glass; patients arms too weak to manage	No fan available on ward No electric bed as promised; uncomfortable bed Wrong diet for 2 days Not being able to see doctor Being dressed in pyjamas that were too tight and gave discomfort Having to use commode when he had diarrhoea and sickness Lack of privacy from disinhibited patients Sleep deprivation due to visitors/TVs/arguments Taken to side room, which felt like being 'locked away in a cupboard' Disabled patient required electric bed but not available Contracted methicillin-resistant *Straphylococcus aureus *in general ward Checking up on feeding and nutrition left to carer/visitors	Inedible food Too few nurses to help with basic care Nurses too rushed to talk to them and find out what kind of help they needed Not enough of the right kind of bed available in hospital Electric bed available but not procured quickly enough No physiotherapy as promised Not enough fans available in hospital Windows jammed shut when fresh air required; no repair/maintenance available	Nurses didn't understand English so communication broke down No apology made after jug of cold water spilt over her Nurses not seeming confident about tracheostomy Nurses struggling to operate suction 'vac' Staff being loud during night time and disturbing sleep No explanations or apologies given about why equipment not available	Nurses not understanding their physical limitations, particularly the need for help with toileting Not understanding that rough handling could cause pain, for instance when hoisting from bed to chair Limited understanding of their fear at being left alone, especially at night Not realizing the importance of getting a special diet

ICU, intensive care unit; NHS, National Health Service.

### Care and support

#### Basic care: attention, rest and personal care

Being able to sleep, rest and recuperate, and receiving sufficient help with mobility and personal care were seen by many respondents as basic requirements for any hospital patient, but more so in their particular case because they had been seriously ill and were therefore weaker than other patients on the ward. When people felt they were unable to sleep and rest, and when they found themselves struggling to cope with mobility and personal care, particularly help with toileting, some became upset. Being left struggling to cope without enough sleep, without the right diet and without the physical strength to manage personal care were stressors identified by many respondents.

Some respondents attributed what they perceived as a lack of basic care to one of two things; nurses on the general ward were 'too busy' to pay them much attention or communication between staff from ICU and the general ward had broken down in some way. When nurses on the general ward seemed reluctant to help them with personal care, some respondents assumed that the handover had gone wrong. When basic needs were overlooked, particularly the need for help with washing and toileting, which had been a fundamental element of ICU care, several respondents described themselves as being shocked and traumatized. Being unable to manage personal care and feeling unclean as a result made several people feel vulnerable; for example, a man aged 47 years admitted for pneumonia explained how difficult it was when he realized he was physically incapable and did not get the help he needed:

'When I first got into the ward I could hardly stand, in fact almost couldn't stand without assistance. And I had an instance where I needed to go to the loo. And they bought a commode for me, which was fine, that was no issue. But because, if you can imagine I couldn't write properly and I couldn't use my arms properly, you can imagine what else I couldn't do. And subsequently they didn't assist me with cleaning up properly, which left me in a less than clean or desirable position.

On another occasion, a day or two later, they brought me the commode again. And because of the circumstance, it spilt on the floor. And the nurses don't clean up spillages, which I find a little bit (...) strange. So it was left to my wife on both of those occasions to clean me properly. And I then determined that it would never happen again. So in future they brought me a wheelchair and took me to the disabled toilets. And I said, "Right, that's fine. Leave me here and I'll, you know, do whatever. And it'll take me as long as I need to get myself sorted". And that to me is very, very difficult to sort of say. And for somebody of my age and my abilities to sort of say that I've got to, or I'm unable to clean myself, is difficult.

And I can only imagine what unfortunately these paraplegics and people feel, where they are totally reliant upon people. I wasn't totally reliant, I was just asking for assistance, and that assistance wasn't always there.' (IC26; man aged 47 years, in ICU with pneumonia.)

Others said their anxiety was made worse by being too weak to attract attention from ward staff. This made them worry about being left alone at night. Several respondents said that they had been told to press a buzzer for help, but they found that they were too weak to do so.

#### Communication breakdowns

Several respondents concluded that medical staff knew very little about them and their capabilities and suggested why they thought this had happened. Some concluded that their medical details could not have been handed over by ICU staff; others deduced that the handover had been 'too medical' to be of practical use to ward staff. One woman, who had had complications during a hysterectomy and later contracted methicillin-resistant *Straphylococcus aureus*, said that the handover must have been inadequate because general ward nurses expected too much of her initially [35].

Some people felt that a lack of communication between ICU and ward staff had led to 'mistakes' or problems with their medication or diets. One man was upset when he did not get 'soft' food he had expected, which was to help him progress to a normal diet:

'They sent me back to the general ward. Now that is a very chastening experience because you're coming out of intensive care where they have looked after you on a one-to-one basis. They then throw you into a general ward, where they don't really know what you've been through. No one actually seemed to be that interested. And you lie there and you think to yourself, "What's happening to me? I came down here, I was feeling really well. I've been in this ward now for two hours and I feel ill again". And it was hard work. And of all the time I was in hospital, which was close on five weeks, I only actually lost my cool one day. And that was the next day in the actual general ward, when nothing, because the operation I had, I hadn't eaten for five weeks, I'd been fed on liquid intravenously.

So when I was discharged from intensive care, the idea was that I would be eating food. I would start off with soup, ice cream, that sort of thing, to get myself back into the habit of eating again. When I got down to the general ward there had been no communication from intensive care to the general ward what I should be doing. They couldn't actually supply the right food for me. So my wife and son had to bring food in for me to eat. Now this was a reversal of trying to get better. This was actually putting me back.' (IC18; man aged 62 years, admitted to ICU with surgical complications.)

#### Adjustment

In ICU patients had received one-to-one care by specially trained nurses, but on a general ward several people said that they felt they were 'one patient amongst many'. For instance one man who was on the general ward for 5 weeks said that his care was adequate but that he had wanted to be asked about how he himself felt 'as a patient':

'You'd gone from this hugely protective critical care to this sort of hit and miss uncoordinated service. There was nothing wrong with the individual care, you know – when somebody came to empty your urine bag, they did it properly, they didn't spill the urine all over the floor. When they came to do your blood pressure they did that properly, but were they really asking me, as a patient, how did I feel? Once in a blue moon the Sister would come and sort of bounce past and say, "How are you today?" And you say, "Well I'm alright". You know, what more can I say?' (ICO3; man aged 66 years, admitted to ICU with surgical complications.)

Other respondents had encountered different attitudes to care on the general ward; some nurses were genuinely caring and approachable, but others gave the impression of wanting to be elsewhere. A 68-year-old man, who needed to be hoisted in and out of his bed, said that although some staff had 'love and care shining from them', others made him feel vulnerable [36].

Writing for critical care nurses, Adam and Osborne [1] noted that patients who have received care in ICU can take up to a year to rebuild their physical strength, and acknowledged that many ward staff do not know this and may see some patients as lazy. This resonated with several accounts in which respondents said that ward staff had taken a stern approach to them when they could not manage self-care. Although one man described how being challenged to do more for himself had pushed him to make a 'significant step forward', others said that they lost their self-respect when they could not cope (see IC26 above).

### Ward organization and environment

Some patients said they had relied on their carers for some time before ward staff noticed that they needed more help with basic care needs. One man's medication was left on his tray table when he couldn't pick anything up. At first he depended on his wife, although 'after a couple of days' he felt that nurses understood his limitations:

'After intensive care we were, I was moved on to a busy, very old fashioned style of ward and it was really like going from the twenty first century to the dark ages. The staff were very willing and very pleasant, but didn't seem to know my limitations as it were.

... On this the second ward, they didn't have that equipment or that knowledge of my symptoms and I think what summed it up for me on the first evening was, the nurse came round to pass out my medication and it was just put on my tray table and I couldn't pick it up. And I didn't know whether I should take it then, I should take it before I went to sleep or whatever. But it was just literally doled out, put on the table. I could talk at the time but not very strongly and had [my wife] not been there, I'm not sure how I would have communicated with the nurse to find out. And it was [my wife] that had to give me the tablets.

To the credit of the nurses on the ward, after a couple of days they realized what I could and couldn't do, so they would feed me my breakfast, etc. But I suppose part of it was pride, you know, I didn't want to ask them because I knew they were busy and yet they would put my breakfast in front of me and I couldn't take it and I couldn't take my drugs, whatever.' (IC35; man aged 33 years, admitted to ICU with Guillain-Barre syndrome.)

Respondents also were upset when equipment they were expecting on the ward did not arrive. Respondents talked about having been promised, but not receiving, equipment such as a tilting bed, a Zimmer frame and a fan. A woman whose husband was transferred to the general ward noticed 'perspiration was pouring off him' and was told that no fans were available, and as a result she experienced 'two hours of total stress knowing that he was hot, all for something so stupid as a fan'.

#### Concerns about the ward environment

Many patients felt unprepared for the busy atmosphere of a general ward and found that they were made anxious and insecure by noise from other patients and their visitors. One man described 'the bedlam' of the general ward, with lights blazing and people arguing 'all over the place'. He and others said that the ward environment hindered their recovery.

Respondents expected the organization of their care to follow their needs, with different levels of care provided for them as they recovered. Although some felt disturbed or fearful of the noise and bustle of the ward, others, who were given more privacy, said they felt isolated and neglected. A woman who had been a nurse on an ICU had appreciated having a single room, but her most pressing need after transfer was attention and care:

'I was put into a side ward because obviously the ward staff thought I needed privacy but no, very upset and very distraught and I had lots of visitors and other staff and I was put into the side ward. But I couldn't even walk, I couldn't get up and sit in the chair by myself. I could feed myself by that point, but I couldn't get to the toilet, I couldn't stand up, I couldn't get to the sink. If my glass was on a trolley at the bottom of the bed I wouldn't have been able to reach that. So it was quite difficult and my relatives saw this and they knew that I'd gone from being in intensive care with my own nurse to suddenly being, alone really. And, you know, they were worried what would happen if I went off in the night, what would happen if nobody could, you know, I couldn't contact anybody so they were worrying about all those kinds of things. And it was quite daunting even for me even though I knew that that would happen, it was still quite daunting and, you know, frightening. And even if you did buzz the nurses, they were obviously busy. They couldn't come as quickly as they could on the intensive care unit, you'd have to wait, which wasn't anybody's fault. But it would have been better if I could have done a bit more for myself.' (IC01; woman aged 41 years, in ICU with complications in pregnancy.)

There were some exceptions to the experiences described above. A few respondents said they had discussed with outreach nurses how they might feel about leaving one-to-one care in ICU before transfer. Several others said they were satisfied with their care in both ICU and the general ward, but two respondents (IC21 and IC22) had spent only a short time on the general ward before either being transferred to another hospital for cancer treatment or being completely discharged. The other respondent who was perfectly satisfied with his care was a 58-year-old man who had been in a motorbike accident and who also said that he could not remember much detail about his time in hospital.

## Discussion

Most respondents in this study had had negative experiences of transferring from ICU to the general ward. Rather than attributing transfer stress to the individual's psychological dislocation, we have considered patients' own perceptions and experiences within the context of their entire narrative, carefully examining what each respondent said and the way their thoughts were expressed. These accounts suggest that not enough care in the ward and the ward environment itself were the main reasons for relocation stress. Many respondents acknowledged that ICU and the ward had different functions, but their experiences on the general ward caused them anxiety and stress, which was not just inevitable anxiety arising from leaving a protected environment.

Apart from the depth and richness of our qualitative interview data, the main strengths of this study lie in the narrative interviews themselves which enable people to give voice to their experiences, allowing greater understanding of how patients make sense of what has happened to them. Our findings go beyond describing what can go wrong for patients and delineate some of the practical causes which may help others identify specific ways of improving care for patients transferring from ICU. The limitations of our study are that we do not have independent information about the facilities, including staff expertise, in the hospitals concerned. Participants had received treatment several months (in some cases years) earlier and we would expect there to be some recall bias. However, the accounts are richly detailed and many of the reported experiences occurred in several accounts, thereby reinforcing the findings.

Recently published recommendations from the NICE Guideline Development Committee on Critical Care used the DIPEx intensive care collection to inform their recommendations. Their recommendations were that the discharge and general ward teams should share responsibility for the care of patients being transferred, and that continuity of care should be considered and if possible supported by a written plan. These recommendations may, if translated into action, address some of the practical shortcomings of care that we discuss here.

As our findings suggest, when people felt that their basic care needs were overlooked and that ongoing support with their recovery was inadequate, they can become demoralized and depressed. Ward follow up, outreach services and other strategies aimed at improving care for ICU patients transferred to the ward are significant developments, but more attention to nursing care on the wards is important. In their guidelines, NICE recommended that staff working with acutely ill patients on general wards should have education and training so that they can recognize and understand the physical, psychological and emotional needs of patients on discharge from the ICU. Our analysis indicates that this would be an invaluable first step toward providing post-ICU patients with the kind of individualized care that many felt they needed.

Changing the pace of the general ward is probably impossible, but our evidence shows that patients' needs differ; some feel that they need quiet and privacy to recuperate and want to be left undisturbed by staff and visitors, whereas others fear being left alone for too long, being hidden behind curtains, or not being able to attract attention. The answer is not simple. Staff on the general ward should be able to elicit information from critical care staff, ensuring that the handover is more than merely clinical, and if possible the patient and the family should be included in decisions about the patient's needs and where in the ward he or she should be put.

## Conclusion

Conceptualizing patients' negative experiences of transferring from intensive care as 'relocation stress' has certainly encouraged policy makers to listen to patients' concerns about their treatment when they leave the ICU. Patients clearly appreciate new strategies of step-down care, although our study suggests that some fundamental practical needs and support are still being overlooked during the transfer process. Not all of the factors associated with relocation stress should be seen as an inevitable consequence of the psychological adjustment involved in transfer from an ICU. There are several aspects of care that deserve further attention from service providers and researchers.

These include the need to train enough ward staff to recognize and cope with the physical and emotional fragility of former ICU patients and their families, and to be prepared to engage with patients and meet their needs.

The term 'relocation stress' has provided a useful framework for discussion of factors that can cause patients distress, but specific improvements will be difficult to make without understanding the concrete causes of the distress. Unless staff in the general ward are specifically trained to respond to the needs of former ICU patients, and are prepared for them to find it difficult to adjust to a less structured environment, patients are likely to continue to experience isolation and fear, which may threaten or delay recovery.

## Key messages

• Patients' anxiety after transferring from ICU should not necessarily be attributed to psychological difficulties arising from leaving a protected environment.

• Former ICU patients and their carers can become distressed and depressed if they feel that they are not receiving well planned care on the general ward.

• Patients and carers are likely to become anxious if the special diets, equipment and support that they need for the next stage of their recovery are not available on the general ward.

• Staff on the general ward should be routinely included in planning care for patients who will shortly be leaving the ICU.

• Handovers from the ICU to the general ward should go beyond the clinical and include details of patients' physical limitations and care needs.

## Abbreviations

DIPEx = Database of Personal Experiences of Health and Illness; ICU = intensive care unit; NHS = National Health Service; NICE = National Institute for Health and Clinical Excellence.

## Competing interests

The authors declare that they have no competing interests.

## Authors' contributions

SP interviewed the patients and analyzed the data together with KF. The intensive care study is part of the DIPEx project. KF drafted the paper; all authors contributed to subsequent drafts and the final version.
